# First application of intraoperative MRI of the liver during ALPPS procedure for colorectal liver metastases

**DOI:** 10.1007/s00423-020-01890-3

**Published:** 2020-05-26

**Authors:** Carina Riediger, Verena Plodeck, Johannes Fritzmann, Alexander Pape, Alexander Kohler, Björn Lachmann, Thea Koch, Jens-Peter Kühn, Ralf-Thorsten Hoffmann, Jürgen Weitz

**Affiliations:** 1 Department of Visceral, Thoracic and Vascular Surgery, University Hospital Carl Gustav Carus Dresden, Technische Universität Dresden, Dresden, Germany; 2 Department of Radiology, University Hospital Carl Gustav Carus Dresden, Technische Universität Dresden, Dresden, Germany; 3 Department of Traumatology and Orthopaedic Surgery, University Hospital Carl Gustav Carus Dresden, Technische Universität Dresden, Dresden, Germany; 4 Department of Anesthesiology and Intensive Care Medicine, University Hospital Carl Gustav Carus, Technische Universität Dresden, Dresden, Germany

**Keywords:** CRLM, Intraoperative MRI, ALPPS

## Abstract

**Purpose:**

Intraoperative detection of intrahepatic lesions can be demanding. The use of preoperative contrast-enhanced magnetic resonance imaging (MRI) or computer tomography (CT) combined with intraoperative ultrasound of the liver is state of the art. Near totally regressed colorectal liver metastases (CRLM) after neoadjuvant chemotherapy or nodules in severely altered liver tissue as steatosis or cirrhosis are often hard to detect during the operative procedure. Especially differentiation between benign atypical nodules and malignant tumors can be very difficult. The intraoperative use of contrast-enhanced ultrasound or intraoperative navigation are helpful tools. However, both methods show relevant limitations.

The use of intraoperative MRI (ioMRI) can overcome this problem. Relevant structures can be marked within the operative site or immediate control of complete tumor resection can be achieved. This might allow immediate surgical optimization in case of failure.

**Methods:**

We report the intraoperative application of ioMRI in a case of a 61-year-old male patient suffering from rectal cancer with 10 synchronous bilobar CRLM who was treated stepwise by multimodal treatment and staged hepatectomy. Intraoperative contrast-enhanced MRI of the liver was used during completion procedure of an extended right hemihepatectomy performed as “Associating Liver Partition and Portal vein Ligation for Staged hepatectomy (ALPPS)”.

**Results:**

ioMRI provided excellent images and showed absence of liver metastases in the liver remnant. Procedure of ioMRI was safe, fast and feasible.

**Conclusion:**

To the best of our knowledge, we describe the first case of intraoperative application of a contrast-enhanced MRI during open liver surgery at the University Hospital of Dresden.

## Introduction

Inspection, palpation, and the use of intraoperative ultrasound (IOUS) are important intraoperative tools to detect and localize liver metastases in open liver surgery that are regularly used.

However, intraoperative localization and detection of liver tumors can be difficult—especially in patients with severe alteration of the liver tissue such as steatosis, steatohepatitis, or liver cirrhosis. Another challenge for intraoperative localization are small nodules as near totally regressed liver metastases after chemotherapy as well as the differentiation between tumor nodules and scar tissue after resection in patients with repeated liver resections or two-staged hepatectomy.

Contrast-enhanced ultrasound, intraoperative use of indocyanine green (ICG) fluorescence imaging, and 3D navigation can help to support the detection of liver tumors. Even though some authors describe the advantages of contrast-enhanced ultrasound of the liver, others show clear limitations of this technique. Intraoperative use of ICG fluorescence imaging in liver surgery can be helpful to detect tumor nodules as well as segment borders within the liver. However, ICG has to be applied a few days before surgery for the detection of tumor nodules [1].

Intraoperative 3D navigation can be helpful, but due to limited experience and variable inaccuracy of this method, the use is actually limited to experimental settings or clinical trials in selected centers.

It has been demonstrated that magnetic resonance imaging (MRI) is an excellent imaging modality of the liver. MRI is sometimes the only modality for the detection and differentiation of liver tumors.

The first intraoperative magnetic resonance imaging (ioMRI) technique was installed in the 1980s [2, 3]. The ioMRI technique was first used in neurosurgical procedures to improve accuracy and safety of surgery [4–6].

The most commonly used application for ioMRI are neurosurgical procedures. However, in recent years, cases and case series of ioMRI for surgery of sarcomas have been published [7, 8].

To the best of our knowledge, this is the first report of an intraoperative use of a MRI during liver resection.

## Description

**Fig. 1:**
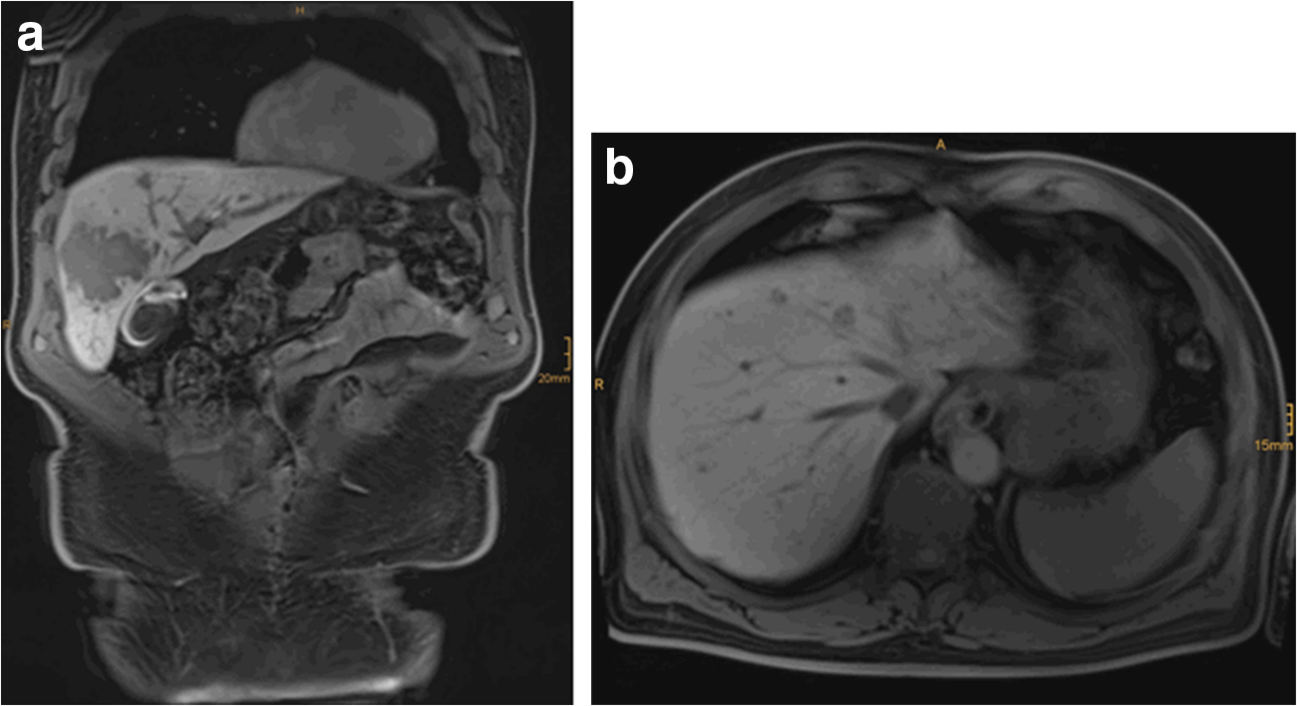
Bilobar CRLM; preoperative contrast-enhanced MRI of the liver, liver specific phase (12.04.2019). **a** coronal plane, **b** transverse plane

We report the case of a 61-year-old male patient suffering from rectal cancer with 10 synchronous bilobar colorectal liver metastases (CRLM) (Fig. 1). After neoadjuvant radio-chemotherapy (5 × 5 GY/5-FU/folinic acid), he received APR in February 2019 in an external tertiary hospital. He was then transferred to our unit for the treatment of liver metastases, and 2-staged liver resection was planned. In April 2019, non-anatomic resection of several metastases of the segments II and III was performed. Metastasis in liver segment IV was not resected due to the infiltration of the middle hepatic vein. Extended right hemihepatectomy was planned and embolization of the right portal vein was performed.

After recovering, the patient received neoadjuvant chemotherapy with oxaliplatin, 5-FU, and folinic acid from 5/2019 until 08/2019.

**Fig. 2:**
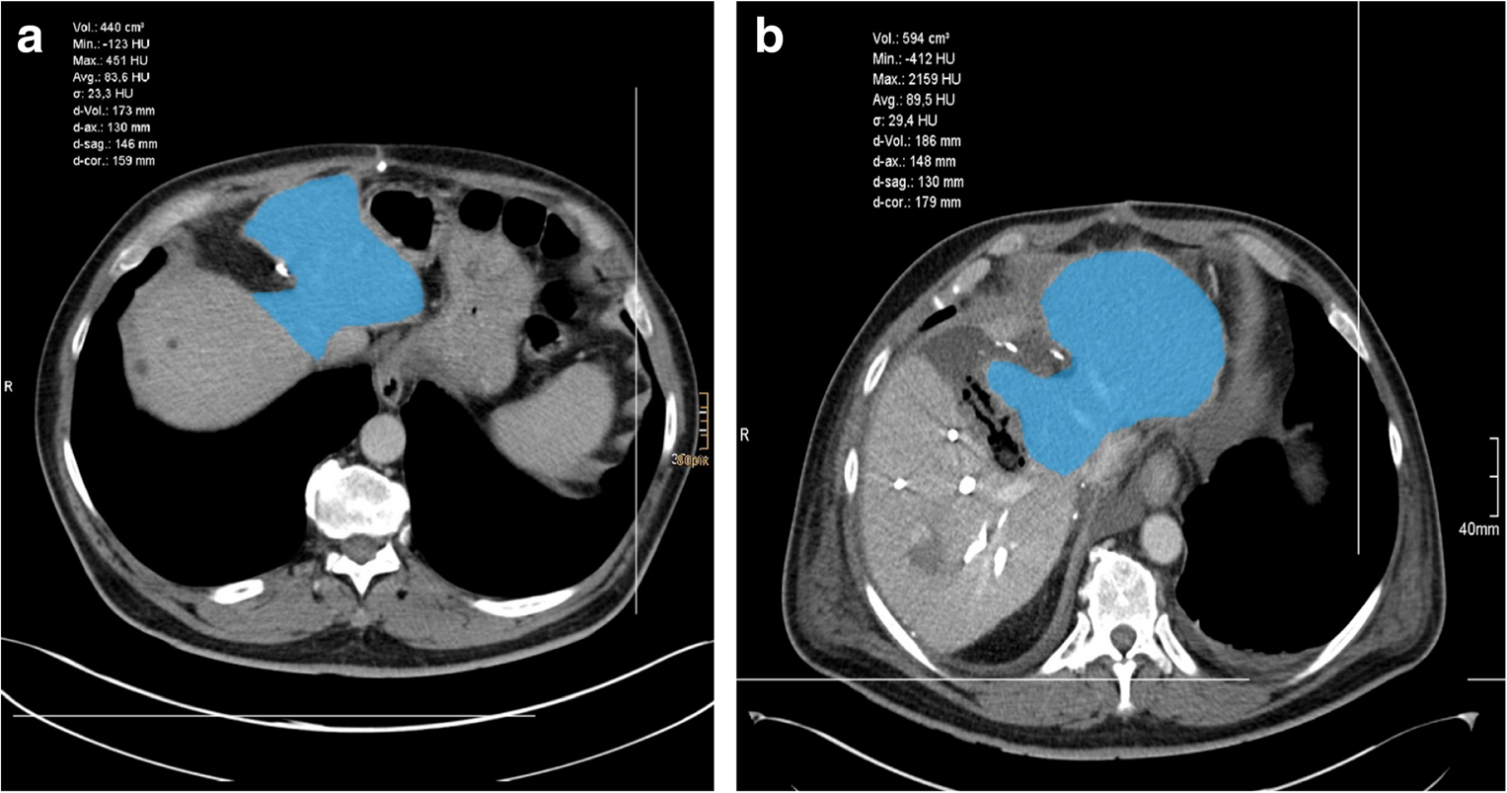
CT-based volumetry of the future liver remnant before (**a**) and 7 days after ALPSS step 1 (17.09.2019) (**b**)

CT scan showed moderate hypertrophy of the liver segments II and III (Fig. 2a). Reoperation was performed on September 11, 2019. Intraoperative evaluation of the liver showed an unfavorable combination of small liver segments II and III and chemotherapy-damaged liver tissue with a high risk for postoperative liver failure in case of one-step extended right hemihepatectomy.

**Fig. 3:**
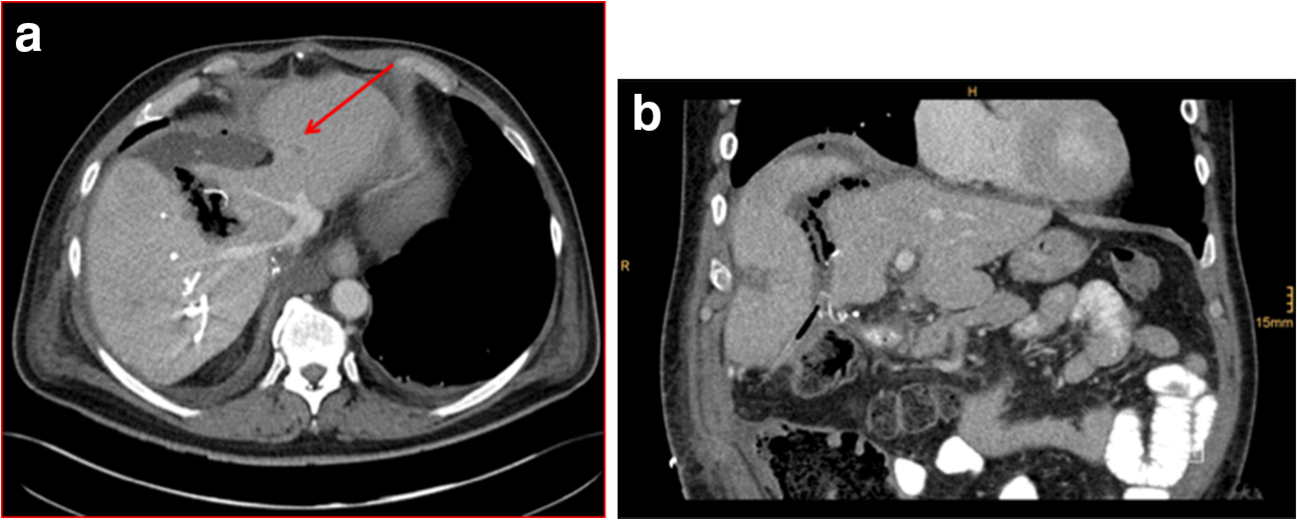
**a** Contrast-enhanced CT scan (venous phase) 7 days after ALPSS step 1 (17.09.2019) in transverse plane showing a small suspicious lesion in segment II (red arrow) (**a**). **b** Coronal view (**b**)

Extended right hemihepatectomy was then performed as “Associating Liver Partition and Portal vein Ligation for Staged hepatectomy (ALPPS)” procedure with parenchymal transection and additional ligation of the right portal vein. Rubber bands around the right hepatic vein, the right hepatic artery, and the right inflow pedicle were left in situ. Control CT scan on the 7th postoperative day (POD) revealed sufficient hypertrophy of segments II and III. Volumetry of the future liver remnant (liver segments I, II, III, and IVa) showed an increase from initially 440 to 594 cm^3^ (Fig. 2a and b). However, a CT scan revealed new suspicion of metastatic recurrence of CRLM after atypical resection in segment II (Fig. 3). To avoid unnecessary additional parenchymal resection, a combination of intraoperative inspection, palpation, ultrasound, and ioMRI was planned. The patient gave informed consent to ioMRI of the liver.

**Fig. 4:**
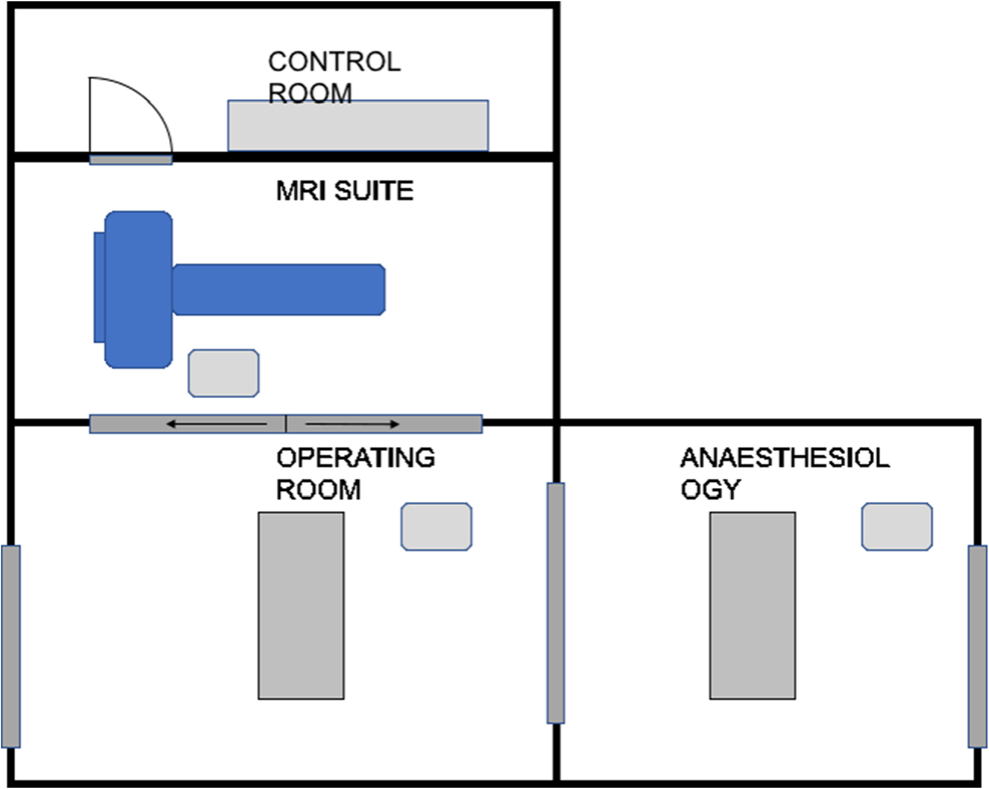
MRI combi suite

Completion surgery was performed in the OR-MRI “Combi Suite” as shown in Fig. 4.

In our hospital, the so-called “Combi Suite” consists of an MRI (3 T, Siemens Skyra, Munich, Germany) door-to-door to a specific operating room (OR). MRI and OR are equipped with specific connectable tables allowing quick patient transfer from OR table to the ioMRI table by a special transfer board without changing patient’s position.

Before using the intraoperative MRI for the patient, cross-training for OR—anesthesiological and MR—personnel has been performed as described by Larson et al. [9].

Furthermore, complete workflow including patient’s transfer from OR to ioMRI as well as the ioMRI was successfully performed with a test person (A.K.). A MRI application specialist guided this procedure.

The completion operation was then performed on September 19, 2019.

Surgery was performed under general anesthesia. After relaparotomy by reopening a Makuuchi incision, adhesions were cleared and palpation and ultrasound of the liver were performed. Nodules of the right lobe were palpable and visible within the ultrasound. In segment III, the suspicious nodule was then marked by a MRI-specific marker that is normally used for breast-specific MRI diagnostics (Bard® DuaLok ™). Rubber bands around the right hepatic vein, right hepatic artery, and right inflow pedicle were left in place. Surgical instruments and retractors were removed from the patient and the operative table.

**Fig. 5:**
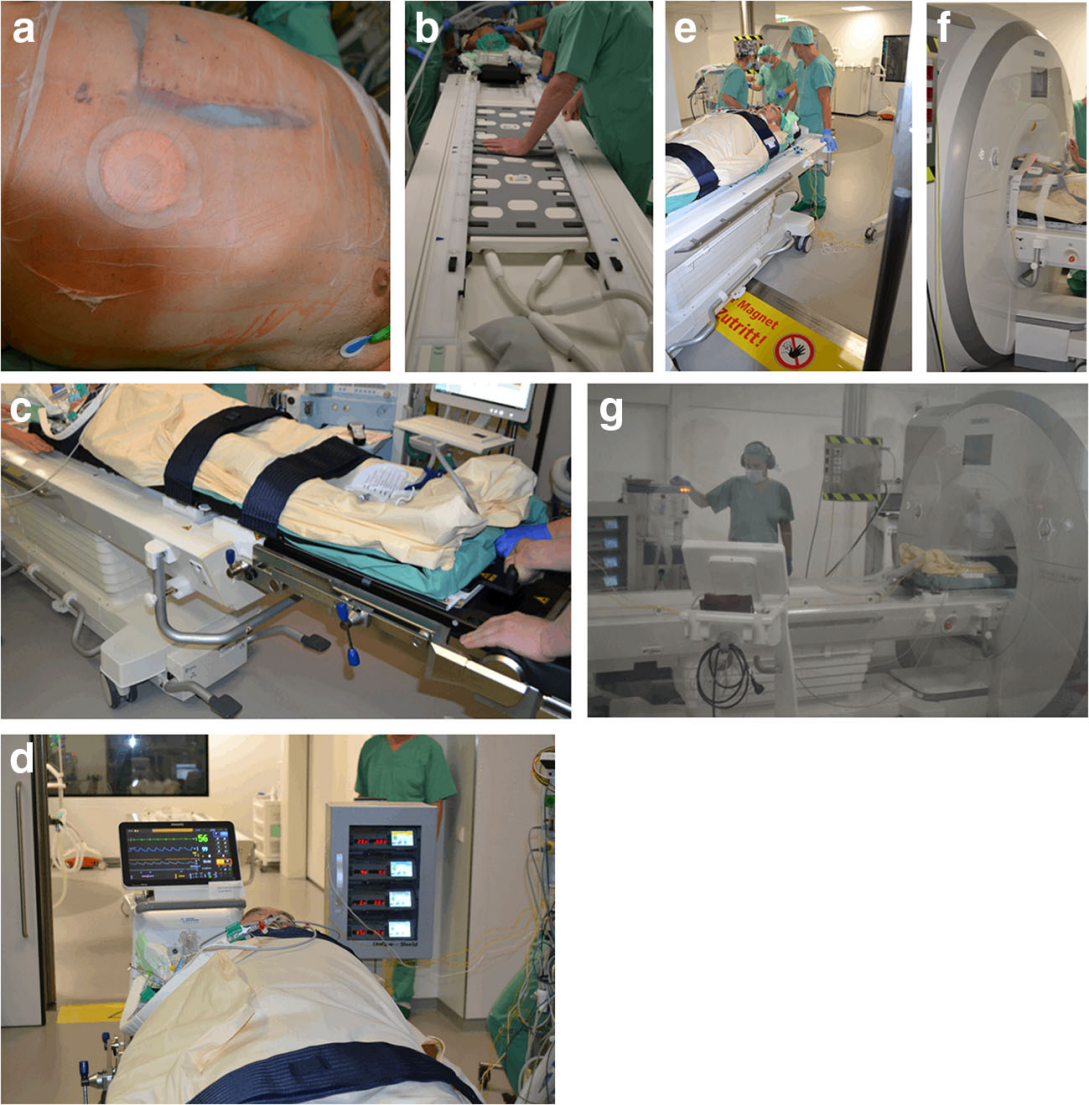
**a** Temporary closure of the abdominal wall by abdominal linen and self-adhesive drape. **b** and **c** Patients transfer from OR table to MRI table by using a special mobile transfer board. **d** View of the OR towards the connected MRI suite showing special MRI-compatibel anesthesiological equipment. **e** and **f** Patients’ transfer into the MRI suite (**e**) and positioning in the MRI with phased array body coil (**f**). **g** View from the MRI control room: anesthesiologist performing apnea episodes during ioMRI

The abdomen was temporarily closed by positioning an abdominal linen under the abdominal wall covered by a sterile, self-adhesive drape/sheet (Fig. 5).

The patient was then transferred to the ioMRI table by using a special mobile transfer board from the operating table. Both OR and MRI tables were connected and locked and the patient was then transferred to the MRI table on the mobile transfer board (Fig. 5b–c).

Anesthesiologic monitoring and equipment were replaced by MRI-compatible devices and disposables. No MRI-compatible equipment, e.g., perfusors, were locked in a MRI-compatible closet while staying connected with the patient (Fig. 5d).

Anesthesia was performed according to the “practice advisory on anesthetic care for magnetic resonance imaging” of the American Society of Anesthesiologists [9].

The whole team completed a security check before the patient entered the ioMRI room. The phased array body coil was placed around the patient (MRI, Siemens, Erlangen, Germany) (Fig. 5e and f).

ioMRI of the liver was performed including the application of a liver-specific contrast medium (8 ml Gadovist®, Bayer Vital GmbH, Leverkusen, Germany) under continuous standardized cardio-respiratory monitoring. No adverse event occurred during the MRI. Apnea episodes of few seconds were performed for better image quality by the anesthesiologist who was in the MRI room during apnea maneuvers (Fig. 5 g).

No heating effect due to abdominal linen was observed.

Immediately after completion of ioMRI, the patient was safely transferred back to the OR and relocated to the OR table.

The total duration of the MRI was 20 min. The transfer time was round trip 15 min.

**Fig. 6:**
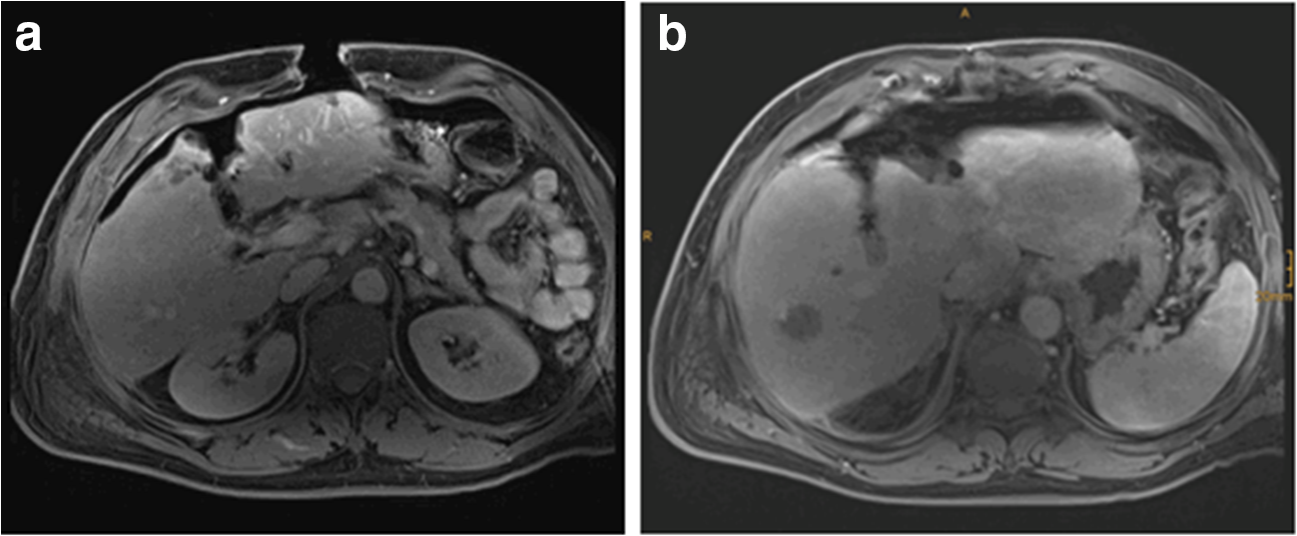
**a**–**b** ioMRI of the liver (19.09.2019) (**a**) and T1-weighted transversal plane after administration of 8 ml Gadovist i.v. (**b**)

Intraoperative MRI provided excellent images which demonstrated no suspicious liver lesions in the liver remnant (Fig. 6a and b). Retrospectively, the lesions seen on CT scan were likely due to the partial volume effect (Fig. 3). Consequently, no additional resection of segment II was necessary allowing completion of the ALPPS procedure as parenchymal sparing as planned. There were no relevant artifacts due to the open abdominal wall, the abdominal linen, or the sterile self-sticking sheet or rubber bands around the hepatic vessels.

Rubber bands were not visible within the MRI images. The breast-specific intrahepatically placed MRI marker was not visible as well. MRI images were immediately analyzed by a radiologist and surgeons.

Subsequently, the operative procedure was completed and extended right hemihepatectomy after ALPSS was performed with a total blood loss of 500 ml without any complication.

The patient’s postoperative course was regular and the patient left the hospital with normal liver function and closed wounds 10 days after the operative procedure, except a self-limitating bile leakage grade A no morbidity occurred.

## Discussion

Improvement of multimodal therapeutic strategies in the treatment of malignant liver tumors as well as the development of surgical techniques and anesthesiological opportunities lead to new aspects in liver surgery.

CRLM shows excellent regression under new chemotherapeutic options in some patients. These so-called disappearing liver metastases (DLM) can hardly be detected intraoperatively, especially in patients showing alterations under chemotherapy as chemotherapy-associated steatohepatitis (CASH) or sinusoidal obstruction syndrome (SOS). In addition, repeated liver resections for CRLM with 3 or 4 repeated liver resections are commonly performed in many patients. In those patients, it can be difficult to differentiate between scar tissue and vital tumor in preoperative and intraoperative imaging.

Inspection, palpation, and the use of IOUS are standard tools for intraoperative localization of liver metastases in open liver surgery. During minimally invasive liver surgery detection has to be performed by inspection and ultrasound as haptic information is reduced.

Intraoperative ultrasound has been described to detect additional lesions not seen in preoperative CT scanning or MRI leading to a change of surgical strategy in 1.4% of cases [10]. The important role of the intraoperative ultrasound to detect lesions that have not been diagnosed in the preoperative MRI was once more confirmed in a recent paper in which is shown that the sensitivity of IOUS was superior to MRI (94.5% vs. 75.1%), with similar specificity. This paper showed that nodules located at the hepatic dome and lesions with mucinous histology are predictive factors for metastases missing at MRI. In this trial, the surgical plan was changed in 23% of patients due to intraoperative detection of new nodules [11].

Another group favors the use of intraoperative real-time virtual sonography with sonazoid enhancement for the detection of liver metastases < 10 mm [12].

However, some authors describe the superiority of preoperative MRI with higher accuracy over intraoperative contrast-enhanced ultrasound in case of DLM [13]. Shiozawa et al. compared preoperative MRI with IOUS/contrast-enhanced ultrasound (CEUS) and reported in their analysis of 133 lesions detection rates of 80% for intraoperative ultrasound, 90.2% for CEUS and 98.5% for endovist-MRI [14].

Other authors recommend intraoperative imaging despite good preoperative CT and MRI or a combination of preoperative hepatocyte-specific MRI and intraoperative contrast-enhanced ultrasound for DLM [15, 16]. Another challenge is the detection and differentiation of hepatocellular carcinomas (HCC) in patients with liver cirrhosis. MRI with hepatocyte-specific contrast medium can be essential for differentiation between HCC and regenerative nodules in liver cirrhosis.

In general, we use preoperative MRI if lesions are not visible in CT and/or ultrasound.

To our knowledge, there are no data available comparing IOUS with ioMRI of the liver. The main indication for ioMRI is tumors that are difficult to detect in ultrasound.

The main entities are hepatocellular carcinomas (HCC) in cirrhotic livers and near-total regressed metastatic lesions under neoadjuvant chemotherapy.

We want to clarify that the use of ioMRI is not used instead of ultrasound, but as an additional tool for patients with metastases that were not visible in preoperative ultrasound or for patients with DLM.

The use of IOUS remains the standard tool for intraoperative imaging during liver surgery. ioMRI is only used in selected patients.

In recent decades, other techniques for intraoperative detection of hepatic lesions were implemented and need to be shortly discussed. There are different methods of intraoperative image guidance as to the application of ICG or intraoperative real-time 3D navigation which can be supportive in detecting intrahepatic lesions. The use of ICG has been shown to detect HCCs as well as CRLM if ICG was applied several days before surgery. However, this method has clear limitations in the detection of small lesions like DLM, especially if located > 5–10 mm distant to the liver surface [1].

Intraoperative 3D navigation is a promising approach—especially for DLM. But due to a lack of accuracy after intraoperative liver shifting this approach is not yet relevant in routine use.

Another more invasive method to detect DLM is the interventional placement of fiducials in each (smaller) metastasis before starting the neoadjuvant chemotherapy to clearly identify the lesions even after macroscopically complete regression [17].

After the first description of interventional and intraoperative MRI suites in the 1980s, the ioMRI technique was mainly applied in neurosurgical procedures [18, 19].

Technical as well as neurosurgical improvement of this technique over the last 30 years led to higher safety and accuracy. Formation of expert and consensus groups as the German Study Group for Intraoperative Magnetic Resonance Imaging or recommendations on anesthetic care for magnetic resonance imaging by the American Society of Anesthesiologists has been elaborated [20].

The general experience and technical developments within the use of intraoperative MRI made this procedure safe and feasible—even in abdominal surgery.

To our knowledge, the intraoperative use of MRI of the liver has not been described before. There are no preceding reports or experiences regarding this technique. No information regarding disturbances of images by open abdomen or intraabdominal material and no information about heating effects on the open abdomen are available.

We could show that ioMRI is safe and feasible even in the patient with a provisory-closed abdomen during liver surgery.

In our opinion, safety is depending on the technical setting. A door-to-door connection between OR and MRI (as seen in our Dresden Combi Suite) is essential for an acceptable time frame and circumstances of sterility.

Careful consideration of high-risk patients is of utmost importance. High-risk patients including patients with critical NYHA state do not qualify for this procedure as well as patients with pacemaker and defibrillator (ICD).

One unknown factor was the temporary closure of the abdominal wall as wet tissues are at risk to develop heating effects and possible disturbances of the MRI images. We showed that temporary closure of the abdominal wall by abdominal linen and self-adhesive sheet was safe without heating or burning problems. Interestingly, no disturbances of the MRI images were seen due to the tissue or the rubber bands. However, the use of a small marker is not sufficient for liver images. Other markers, e.g., liquid-containing markers, need to be tested in the future. Images of the ioMRI were of high quality and influenced further surgical procedure: in our patient, additional liver resection was avoided by the use of the ioMRI.

It has to be considered that there are some limitations for ioMRI during liver surgery:

Patient-specific limitations for ioMRI during liver surgery are MRI-incompatible implants such as pacemaker and ICD and any other metallic foreign body. Despite the fact that there is no weight limit for the ioMRI used in our hospital, the body size and girth/circumference of patient’s body is an important limitation. The patient has to fit in the bore. In the ioMRI used in this case, the maximum diameter of the bore is 70 cm.

Besides patient-specific limitations, major concerns are the additional costs and limited availability of the ioMRI.

The costs of the ioMRI-imaging are about 750, − euros/h including the imaging and radiological personnel. In this case, the additional costs are calculated with about 350, − euros. Noteworthy, as the Combi Suite—MRI is also used for non-intraoperative MRI, the costs of a regular MRI and the ioMRI are identical. There are no additional costs for patient transport as the ioMRI and the OR are designed as Combi Suite and both rooms are connected door-to-door. Additional costs for the occupation of OR and surgical and anesthesiological personnel during ioMRI-procedure cannot be provided in a valid calculation.

## Conclusion

To our knowledge, we describe the first intraoperative MRI during an open liver resection. The use of ioMRI of the liver is safe and feasible and provides excellent images of the liver.

The application of intraoperative liver MRI is not necessary for routine hepatectomies but can provide excellent information in selected patients. In our opinion, the use of intraoperative liver MRI is promising in detecting sonographic occult liver tumors. Moreover, documentation of complete resection of hepatic tumors immediately after resection can avoid R1-/R2-situations and early reoperation and should reduce the rate of intrahepatic tumor recurrence. However, this needs to be proven in further studies.
